# Magnetic Nanoparticles Functionalized Few-Mode-Fiber-Based Plasmonic Vector Magnetometer

**DOI:** 10.3390/nano9050785

**Published:** 2019-05-22

**Authors:** Yaofei Chen, Weiting Sun, Yaxin Zhang, Guishi Liu, Yunhan Luo, Jiangli Dong, Yongchun Zhong, Wenguo Zhu, Jianhui Yu, Zhe Chen

**Affiliations:** 1 Key Laboratory of Optoelectronic Information and Sensing Technologies of Guangdong Higher Education Institutes, Jinan University, Guangzhou 510632, China; chenyaofei@jnu.edu.cn (Y.C.); guishiliu@163.com (G.L.); jldong@jnu.edu.cn (J.D.); ychzhong@163.com (Y.Z.); zhuwg88@163.com (W.Z.); kensomyu@gmail.com (J.Y.); thzhechen@163.com (Z.C.); 2 Department of Optoelectronic Engineering, College of Science and Engineering, Jinan University, Guangzhou 510632, China; sunweiting130@126.com (W.S.); yaxinzhang98@163.com (Y.Z.)

**Keywords:** magnetic nanoparticles, few-mode fiber, surface plasmonic resonance, magnetic vector

## Abstract

In this work, we demonstrate a highly-sensitive vector magnetometer based on a few-mode-fiber-based surface plasmon resonance (SPR) sensor functionalized by magnetic nanoparticles (MNPs) in liquid. To fabricate the sensor, a few-mode fiber is side-polished and coated with a gold film, forming an SPR sensor that is highly sensitive to the surrounding refractive index. The vector magnetometer operates based on the mechanism whereby the intensity and orientation of an external magnetic field alters the anisotropic aggregation of the MNPs and thus the refractive index around the fiber SPR device. This, in turn, shifts the resonance wavelength of the surface plasmon. Experimental results show the proposed sensor is very sensitive to magnetic-field intensity and orientation (0.692 nm/Oe and −11.917 nm/°, respectively). These remarkable sensitivities to both magnetic-field intensity and orientation mean that the proposed sensor can be used in applications to detect weak magnetic-field vectors.

## 1. Introduction

Magnetic sensing is important in the industry, power transmission, military, etc. In recent years, optical-fiber magnetic-field sensors, integrated with magnetic nanoparticles (MNPs) in liquid that is called magnetic fluids (MFs) or ferrofluids, have attracted considerable attention because of their ease of fabrication, high sensitivity and low cost [1]. MFs, a type of paramagnetic material, consist of MNPs uniformly dispersed in a base liquid with the aid of surfactant [2]. When exposed to an applied magnetic field, the MNPs realign and form a chain- or cluster-like structure [3], endowing the MF with various outstanding magneto-optical properties, including a magneto-controllable refractive index (RI), absorption coefficient, birefringence, and so on [4]. Taking advantage of these properties, researchers have proposed diverse magnetic-field sensors based on MFs and various fiber-optical structures or schemes. Some examples include tapered two-mode fiber interferometers [5], Sagnac interferometers [6], multimode interferometers [7], whispering gallery mode resonators [8,9] or interferometers [10,11], microfiber-based interferometers [12,13], two-core fiber-based interferometers [14], photonic crystal fibers [15,16,17] and asymmetric-tapered fiber [18]. Furthermore, based on the mechanism whereby protein binding can tailor the response to a magnetic field, these devices have recently been used to measure protein concentrations [19,20], further extending the range of applications to biosensing. However, most of these sensors were designed to detect the magnetic intensity while ignoring the magnetic orientation, due to the lack of exploration on the microstructure of MF around the optical fiber.

In 2016, Zhang et al. [21] demonstrated the phenomenon that a magnetic field could induce a non-uniform distribution of MNPs, and thus a non-uniform distribution of RI, around an optical fiber. This RI distribution can be tuned by modulating either the intensity or the orientation of the magnetic field. This work revealed a new way to detect both the magnitude and orientation of a magnetic field. By creating a non-circularly-symmetric light-field in a fiber, Yin et al. (2017) [22,23] developed two MF-based magnetic-vector sensors by sandwiching a piece of double-clad photonic-crystal-fiber or a thin core fiber between two single-mode fibers with a lateral offset. In 2018, based on a similar mechanism, magnetic-vector sensing was achieved by Layeghi et al., who used a tapered Hi-Bi fiber inserted in a fiber loop mirror [24]. In 2019, Cui et al. [25] and Lu et al. [26] respectively proposed using a single-mode-fiber fused with capillary structure and an excessively tilted fiber grating to realize the sensing to the magnetic vector. Note that in the above cases, the used fibers still remained in a cylindrically symmetric structure. In 2018, Violakis et al. demonstrated that an MF-encapsulated, D-shaped fiber (a non-circularly-symmetric structure) can also respond to the changes of an external magnetic field’s direction [27], which further paved the ways to use an asymmetric fiber structure. Although a number of fiber-based magnetic-vector sensors have been developed, the simultaneous high sensitivity to both magnetic intensity and orientation still remains as a challenging issue.

Surface plasmon resonance (SPR) sensors are typically constructed by coating a metal film over a dielectric substrate, and they are powerful tools for detecting tiny changes in the RI over a metal surface thanks to their high RI sensitivities, which even can exceed 10^4^ nm per refractive index unit (RIU) around the RI of 1.33. Therefore, combining MFs with SPR technology could provide a scheme for highly-sensitive magnetic-field sensing, and several works in that direction have already been published [21,28,29,30,31,32]. Ying et al. made a numerical study of the magnetic-field response based on a Kretschmann prism SPR configuration [28] which, unfortunately, is limited in practical use because of its bulky size. Weng et al. [29] and Liu et al. [30] theoretically investigated magnetic-field sensors based on side-hole fiber SPRs and D-shaped photonic-crystal-fiber SPRs, respectively. Schwendtner et al. experimentally boosted the sensitivity up to ~1 nm/Oe by using a tapered fiber as an SPR substrate [31]. Unfortunately, these SPR-based MF sensors were only proposed to detect magnetic field intensity, but not orientation. Later, Zhang et al. demonstrated a plasmonic fiber-optic vector magnetometer based on directional scattering between polarized plasmon waves and ferro-magnetic nanoparticles, and achieved the sensitivities of 0.18 nm/Oe and 2 nm/° to intensity and orientation, respectively [21]. Recently, we proposed a fiber SPR sensor, based on side-polished multimode fiber, for the sensing to magnetic vector, and further improved the sensitivities to 0.599 nm/Oe and −5.63 nm/° [33]. However, to realize the sensing to a weaker magnetic field, the magnetic fields’ sensitivities to orientation and intensity need further development.

In this paper, aiming to develop a higher sensitivity magnetometer with the ability of simultaneous sensing to the magnetic intensity and orientation, we propose and investigate a plasmonic vector magnetometer based on a side-polished few-mode-fiber (FMF) functionalized by MNPs. This customized SPR device’s high sensitivity to RI means this sensor is highly-sensitive to magnetic-field intensity (up to 0.692 nm/Oe). In addition, the non-circularly-symmetric geometry of the side-polished fiber and the non-uniform distribution of the MF around the fiber together allow the sensor to sense the magnetic field’s orientation with the high sensitivity of −11.917 nm/°. The excellent characteristics of this sensor and its operating mechanism are comprehensively analyzed herein, offering an effective solution for high-sensitivity magnetic-vector sensing.

## 2. Principles and Structure

**Figure 1:**
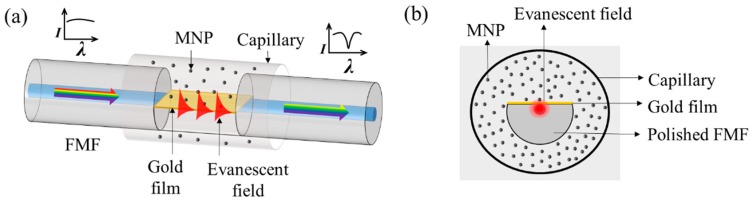
Schematic diagrams of (**a**) the proposed sensor and (**b**) a cross section of the sensor. A broadband incoming light from the left is partially absorbed due to the SPR, producing the notched spectrum of the outgoing spectrum on the right end.

A schematic diagram of the proposed sensor is shown in Figure 1. In this work, the side-polished fiber was made from an FMF. Its core diameter is less than that of a multimode fiber but greater than that of a single-mode fiber, which alleviates the problems of low sensitivity and low signal-to-noise ratio that plagues SPR sensors based on side-polished multi-mode and single-mode fibers [34,35]. The sensor consists of a side-polished FMF (OFS Fitel, LLC) coated with a gold film and immersed in MF (EMG 605, Ferrotec. Inc., Santa Clara, USA) which is sealed in a capillary tube. The MNPs with the material of Fe_3_O_4_ that evenly dispersed in the MF have the average diameter of ~10 nm and the volume concentration of 3.9%, i.e., the ratio of the total volume of MNPs to the volume of MF is 3.9%. When the light in the FMF propagates over the gold-coated region, the p-polarized component of the resonance wavelength will couple with a surface plasmon wave in the gold film, if their wave vectors match. As a result, the transmittance spectrum will feature a narrow-band absorption at the resonance wavelength, which is extremely sensitive to the surrounding RI. Conversely, the RI of the surrounding MF depends on the applied magnetic field [36], so the resonance wavelength depends strongly on the external magnetic field. Therefore, magnetic-field sensing can be implemented by monitoring the resonance wavelength.

**Figure 2:**
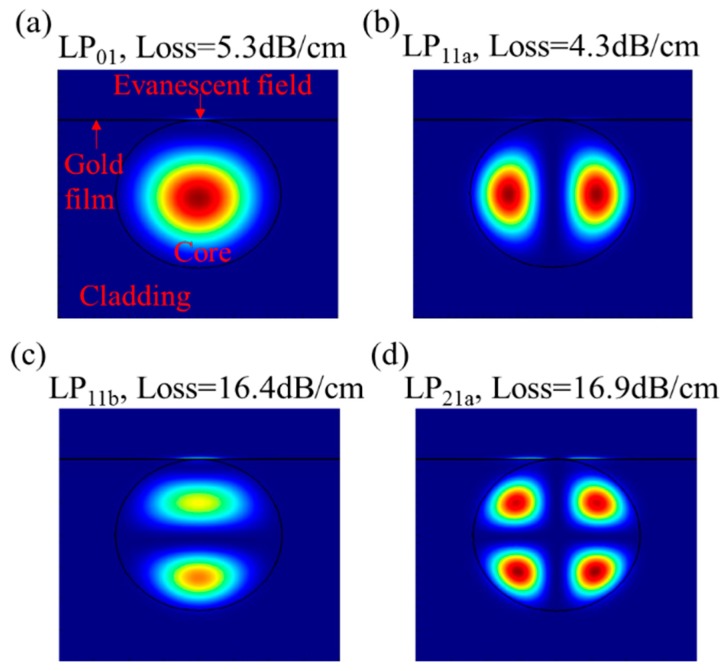
Field distributions in the fiber for modes (**a**) LP_01_, (**b**) LP_11a_, (**c**) LP_11b_, and (**d**) LP_21a_ at 650 nm.

A mode solver (Mode Solution, Lumerical Solutions, Inc.) was used to simulate the mode field distribution. For these simulations, the FMF core and cladding diameters are 19 and 125 μm, respectively; the RIs of the cladding and the FMF core are 1.444 and 1.449, respectively; the RI of the surrounding MF, under zero magnetic field, is 1.385, which is calibrated by a homemade, prism-based SPR testing system; and the thickness of the side-polished FMF is 71 μm, which means that the fiber is polished into the core section. Figure 2 shows the field distributions and the propagation losses of modes LP_01_, LP_11a_, LP_11b_, and LP_21a_ at the wavelength of 650 nm. A strong evanescent field is seen over the gold film surface, indicating SPR excitation.

**Figure 3:**
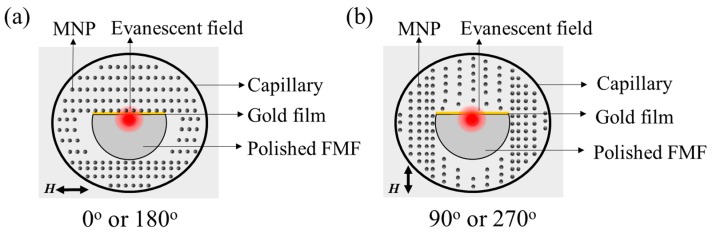
Schematic diagrams of the magnetic nanoparticles (MNPs) distribution around the side-polished fiber, when the external magnetic field is (**a**) parallel and (**b**) perpendicular to the flat surface of the side-polished fiber.

As the evanescent field, which interacts with the surrounding MF, only exists near the gold film surface, the MF microstructure, which is related to the RI over the gold surface, determines the sensor performance. When exposed to an applied magnetic field, the MNPs in the MF become magnetic dipoles and aggregate, forming stable chain-like structures oriented along the applied magnetic field direction [3]. However, the optical fiber in the MF destroys the original equilibrium state, forcing the MNPs to realign and form a new distribution around the fiber [23]. Moreover, the MNPs aggregate in an anisotropic manner: when the fiber surface is parallel (perpendicular) to the magnetic-field direction, they tend to gather to (depart from) the fiber surface [21,22]. For our situation, where the fiber has a D-shaped cross section, Figure 3 shows the state of MNP aggregation around the fiber, which is predicted depending on the published works [21,22,23]. The comparison of Figure 3a,b shows that the aggregation state of the MNFs over the gold film depends on the magnetic field orientation. First, the chain-like structures realign along the magnetic field direction. Second, when the magnetic field direction changes from parallel to perpendicular (relative to the side-polished surface), some of the MNPs depart from the surface of the gold film, leaving fewer MNPs to interact with the evanescent field. The orientation-dependent distribution of MNPs around the fiber is the basis for sensing the orientation of the magnetic-field vector.

## 3. Fabrication and Characterization

A piece of FMF was first coarsely polished by a homemade wheel-polishing system for ~4 min, and then finely polished for ~150 min to obtain the desired residual thickness of the polished fiber. The polished fiber profile was characterized with a microscope (Zeiss Axio Scope A1); the results show that the polished region is ~6 mm long, and the residual thickness of the polished region is ~71 μm.

**Figure 4:**
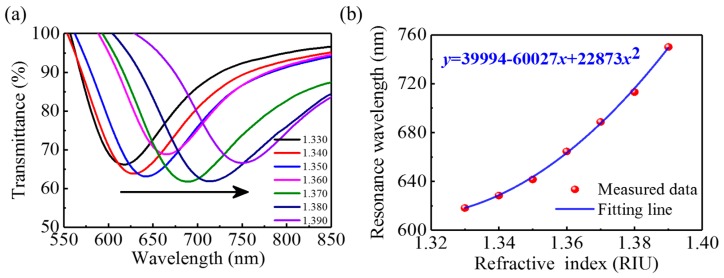
(**a**) Spectral response of the surface plasmon resonance (SPR) sensor to refractive index (RI). (**b**) Measured resonance wavelength as a function of RI.

To fabricate an FMF-based SPR sensor, a chromium adhesion layer (~5 nm) and a gold film (~50 nm) were successively vacuum evaporated onto the surface of the polished fiber. The fabricated SPR sensor’s sensitivity to the RI was characterized by immersing the sensing section into solutions with different RIs. Figure 4a shows the spectral transmittance of the sensor immersed in various RI solutions. With increasing RI, the resonance spectrum shifts to longer wavelengths because the wave-vector-matching condition between the light and the surface plasmon wave depends on the RI [37]. The resonance wavelength varies nonlinearly with RI over the entire range of 1.33~1.39, as shown in Figure 4b. The blue curve in Figure 4b is a fit to a quadratic polynomial. At the RI of MF (~1.385), the SPR sensor’s sensitivity is estimated to be 3693 nm/RIU. The RI sensitivity of the FMF-based SPR sensor exceeds those of optical-fiber RI sensors based on mode interference [38], long-period gratings [39], Fabry–Perot cavities [40], etc., laying a solid foundation for high-sensitivity sensing of magnetic fields.

**Figure 5:**
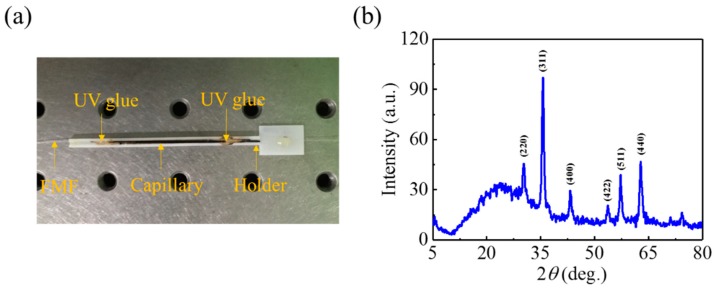
(**a**) Photograph of the fabricated sensor on an optical table. (**b**) XRD pattern for the Fe_3_O_4_ MNPs.

After characterizing the RI sensing performance, the SPR-based magnetic-field sensor was fabricated by packaging the MF around the side-polished fiber. The side-polished fiber section was first fed into the center of a 30-mm-long capillary tube with an inner (outer) diameter of 0.5 (1.0) mm. The capillary containing the fiber was embedded into a holder consisting of a rectangular groove. Next, the MF was inserted into the capillary using capillary force. Finally, the capillary was sealed by applying UV glue at both ends, completing sensor fabrication. Figure 5a shows a photograph of the fabricated sensor, where the black region is the MF-encapsulated in the capillary. The X-ray diffraction (XRD) characterization for the Fe_3_O_4_ MNPs was conducted and the XRD pattern is shown in Figure 5b. As we can see, the peaks shown in the pattern agrees well with the typical peaks of Fe_3_O_4_ standard diffraction at 30.1°, 35.5°, 43.1°, 53.4°, 57.0° and 62.6°, corresponding to the (220), (311), (400), (422), (511) and (440) crystal planes, respectively.

## 4. Experiments and Results

### 4.1. Experimental Setup

**Figure 6:**
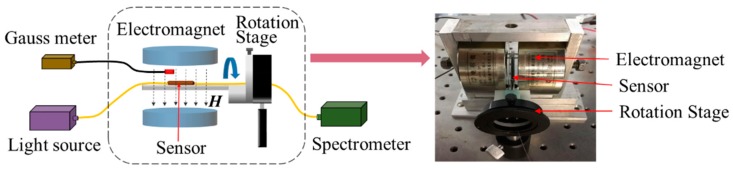
Schematic diagram of experimental setup and photograph of main components, including electromagnet, sensor and rotation stage.

Figure 6 shows a schematic diagram of the experimental setup. A tungsten-halogen lamp broadband light source (AvaLight-HAL-(S)-Mini, Apeldoorn, Netherlands) is coupled into the fiber sensor, and a spectrometer (AvaSpec-ULS2048XL, China) is used to record the transmission spectra. An electromagnet generates the applied magnetic field, which is perpendicular to the fiber axis. The intensity is monitored in real time by a gauss meter. The magnetic-field intensity can be tuned by adjusting the applied voltage. The sensor is fixed onto a rotation stage to allow tuning its orientation with the applied magnetic field.

### 4.2. Response to Magnetic-Field Orientation

To characterize the sensor’s response to the magnetic-field direction, it was rotated from 0° to 360° in 4° increment while the magnetic field’s intensity was fixed. Here the orientation angle is the one between the magnetic-field direction and the polished surface. Therefore, 0° and 180° (90° and 270°) indicate the magnetic field is parallel (perpendicular) to the flat surface of the side-polished fiber, as shown in Figure 3a,b.

**Figure 7:**
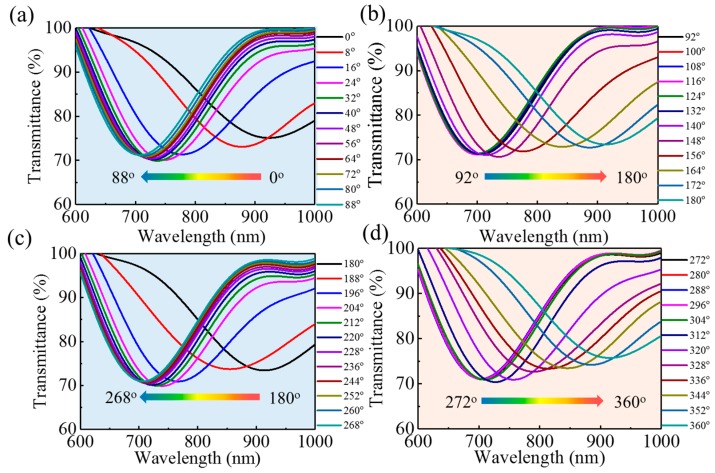
Spectral response of sensor to orientation of magnetic field. The magnetic-field intensity is fixed at 300 Oe, while the orientation is changed (**a**) from 0° to 88°, (**b**) from 92° to 180°, (**c**) from 180° to 268°, and (**d**) from 272° to 360°.

First, the magnetic intensity was fixed at 300 Oe. Upon rotating the sensor counterclockwise (i.e., changing the relative orientation between magnetic field and sensor), the transmission spectrum blueshifts and redshifts repeatedly, indicating the sensor is sensitive to the applied magnetic field direction. Figure 7 shows the spectral response to the relative orientation of the magnetic field: the transmission dip, due to the SPR, blueshifts as the orientation angle goes from 0° to 90° and from 180° to 270°; On the contrary, the dip in transmission redshifts when the orientation angle goes from 90° to 180° and from 270° to 360°.

**Figure 8:**
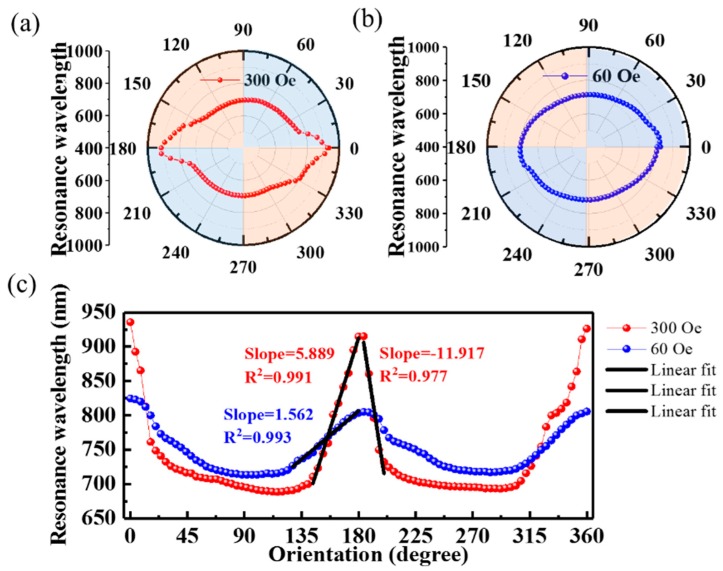
Resonance wavelength as a function of magnetic-field orientation plotted in (**a**,**b**) polar coordinates and (**c**) Cartesian coordinates. The magnetic field intensity is fixed at 300 Oe (red dots) and 60 Oe (blue dots).

Figure 8a shows the resonance wavelengths at different orientations in a polar coordinate system. The noncircular curve indicates the orientation-dependent sensor response. The resonance wavelength depends on the orientation angle because the MNPs anisotropic aggregation around the side-polished fiber [22,23]: when the magnetic field goes from parallel to perpendicular with respect to the gold film, the MNPs concentration over the gold film gradually decreases, as illustrated in Figure 3. As the RI of the MF correlates positively with the concentration of MNPs, the RI of the MF over the gold film will reach its maximum (minimum) at 0° and 90° (180° and 270°). Therefore, four extreme points appear in the resonance wavelength curve versus orientation angle, as shown in Figure 8a, and the resonance wavelength changes monotonically from one maximum (minimum) to the adjacent minimum (maximum) upon monotonically varying the orientation. In addition, Figure 8a shows that the change around the maxima (0° and 180°) is sharper than that around the minima (90° and 270°), indicating a deviation from parallel orientation induces a larger change in the RI over the gold film than it does from a perpendicular orientation.

Another series of tests to characterize the sensor’s orientation response were conducted by the same way, but with a magnetic-field intensity of 60 Oe. Figure 8b shows the resonance wavelength as a function of orientation angle, which presents a similar trend to a magnetic-field intensity of 300 Oe (cf. Figure 8a). However, the curve is more circular, indicating that the lower magnetic-field intensity weakens the orientation dependence, which arises from the smaller change in the RI of the MF at the lower magnetic-field intensity.

To quantitatively assess the orientation response, we plot, in Figure 8c, the resonance wavelength versus orientation angle in Cartesian coordinates and show the corresponding linear fits in the linear regions. Within the range of 144°~180°, the sensitivities to orientation are 5.889 nm/° and 1.562 nm/° for 300 and 60 Oe respectively, indicating that the sensor is more sensitive to orientation at a higher magnetic-field-intensity. Within the range of 184°~200° at 300 Oe, we can achieve the maximal sensitivity of −11.917 nm/°, which is much higher than the 2 nm/° and −5.63 nm/° obtained by using the SPR scheme based on a tilted fiber-Bragg grating [21] and a side-polished multimode fiber [33], respectively. This advantage can be attributed to the non-circularly-symmetric cross section of the side-polished fiber and the smaller core diameter of FMF than that of multimode fiber.

### 4.3. Response to Magnetic-Field Intensity

**Figure 9:**
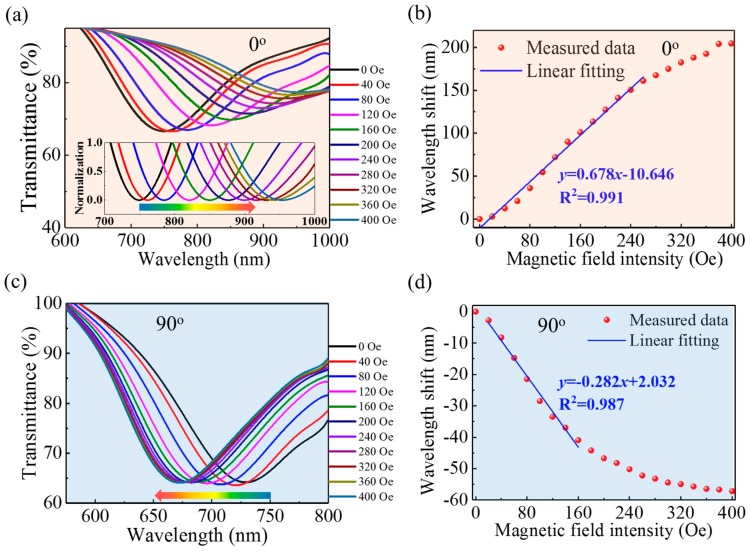
(**a**,**c**) Spectra for different magnetic-field intensities and (**b**,**d**) corresponding resonance wavelength as a function of magnetic-field intensity. Panels (a,b) correspond to the orientation angle of 0°, and panels (c,d) correspond to the orientation angle of 90°. The inset in (a) shows the normalized transmittance spectra near the resonance wavelengths.

The sensor’s response to the magnetic-field intensity was characterized by gradually changing the intensity from 0 to 400 Oe while keeping the orientation fixed. During the measurements, the orientation was fixed at two specific angles (i.e., 0° and 90°), corresponding to the magnetic field being parallel and perpendicular, respectively, to the flat surface of the polished fiber. Figure 9 shows the transmission spectra for several magnetic-field intensities and the resonance wavelength as a function of magnetic-field intensity.

As shown in Figure 9, the transmission dip, due to the SPR, redshifts (blueshifts) with the increase of magnetic-field intensity when the orientation angle is 0° (90°). This indicates the change in the RI over the gold film for the parallel orientation is opposite that for the perpendicular orientation, which is attributed to the MNPs anisotropic aggregation around the side-polished fiber: as illustrated in Figure 3, the MNPs approach (or distance themselves from) the gold film when an external magnetic field is applied parallel (or perpendicular) to the surface of the gold film. Moreover, increasing the magnetic-field intensity further enhances this gathering or evacuation of MNPs, resulting in a further increase or decrease of the RI over the gold film. Figure 9b,d show the resonance wavelength as a function of magnetic-field intensity for parallel and perpendicular orientation, respectively. Overall, the wavelength is nonlinear in magnetic-field intensity, which is mainly attributed to (i) the nonlinear dependence of the RI of the MF on magnetic field, which usually follows a Langevin function [41]; and (ii) the nonlinear sensitivity to RI of the SPR sensor, as shown in Figure 4. Nevertheless, the linear regions were fitted to characterize the sensitivity of the device to magnetic-field intensity. For 0° and 90°, the sensitivity to magnetic-field intensity is 0.692 nm/Oe (0~220 Oe) and −0.282 nm/Oe (20~160 Oe), respectively.

To make a clear comparison, we summarized the relevant-published works that use SPR and MF for magnetic field sensing in Table 1. As we can see, the sensitivity to magnetic-field-intensity achieved in this paper is competitive with the most reported results. More importantly, the employing of side-polished FMF, which has a non-circularly-symmetric geometry and a relatively-smaller core diameter compared to multimode fibers, makes the proposed senor possess the highest sensitivity to magnetic-field-orientation.

### 4.4. Control Experiment

**Figure 10:**
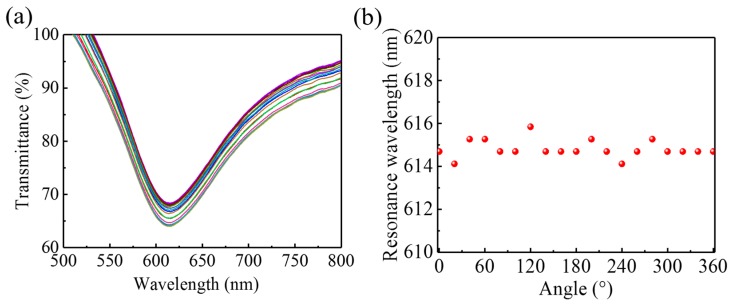
(**a**) Transmission spectra for different orientations and with no magnetic field, and (**b**) resonance wavelength as a function of rotation angle.

In the experiments, the applied magnetic-field’s relative orientation is modified by rotating the sensor, which inevitably twists the optical fiber. To nullify any effect from a twisted fiber, which may cause a further change in the fiber’s state of polarization and birefringence [43,44], we did a control experiment in which the MF was replaced with distilled water and the sensor was rotated over several full rotations. Figure 10a shows the transmission spectra for several rotation angles. Although the spectrum fluctuates slightly, no clear shift in the transmission dip appears. The corresponding resonance wavelengths are presented in Figure 10b. The largest shift in the resonance wavelength induced by rotation is only ~2 nm, which is much less than what occurs when the SPR sensor is immersed in the MF, as shown in Figure 7 and Figure 8. Therefore, we conclude that, in these experiments, the shift in resonance wavelength is induced by the magnetic-field intensity and the MF’s orientation-dependent optical properties instead of by any twisting of the fiber that may be induced by rotating the sensor.

### 4.5. Discussion

We have demonstrated a highly sensitive device to detect magnetic-field vectors. The device is based on a side-polished-FMF SPR immersed in an MF. By exploiting the intrinsic high sensitivity of the SPR to the RI and the strong magneto-optical effect in the MF, the proposed sensor is highly sensitive to magnetic-field intensity. Additionally, the nonsymmetrical configuration of the side-polished fiber and the anisotropic aggregation of MNPs around the fiber make the sensor highly-sensitive to the magnetic field’s relative orientation. At a fixed magnetic-field intensity, the RI of the MF in the vicinity of the side-polished surface is maximized when the magnetic field is parallel to the surface. Moreover, in the parallel orientation, the RI of the MF increases with increasing magnetic-field intensity, causing the resonance wavelength to redshift. On the contrary, in the perpendicular orientation, the resonance wavelength blueshifts with increasing of magnetic-field intensity because the RI of the MF is smaller in this orientation.

The measurement resolution is calculated using *R* = *σ*/*S* [45], where *σ* is the standard deviation of the output noise, measured multiple times under the same conditions, and *S* is the sensor’s sensitivity. In the present work, *σ* = 0.156 nm, which is determined mainly by the spectrometer’s performance. As a result, the sensor resolution is calculated to be 0.225 Oe and 0.013° for magnetic-field intensity and orientation, respectively.

A common problem with such MF-based sensors is the temperature’s effect on their performance, which is due to the thermo-optical effect (about −10^−4^ RIU/°C depending on the concentration of MNPs) [41]. In our case, the RI sensitivity of the SPR sensor was determined to be 3693 nm/RIU for a RI ~1.385 (i.e., the RI of the MF under zero magnetic field). Therefore, we calculate the sensor’s temperature sensitivity to be −0.369 nm/°C. Then, considering the measured sensitivities of 0.692 nm/Oe (sensitivity to magnetic-field intensity) and −11.917 nm/° (sensitivity to magnetic-field orientation), the error induced by the temperature fluctuation will be 0.533 Oe/°C and 0.031°/°C for magnetic-field intensity and orientation, respectively. The temperature sensitivity is less than the magnetic-field sensitivity, indicating that temperature is a minor factor in determining the proposed sensor’s performance. Nevertheless, a method to eliminate or alleviate the temperature effect is desired and will be the subject of a future presentation.

## 5. Conclusions

In summary, we proposed and investigated an optical-fiber-based magnetic-vector sensor that offers high sensitivity to both the magnetic-field intensity and orientation. The proposed sensor consists of an SPR sensor based on a side-polished FMF functionalized by immersion in an MF. Experiments show that the maximal sensitivities of the proposed sensor can reach as high as 0.692 nm/Oe (to magnetic-field-intensity) and −11.917 nm/° (to magnetic-field-orientation). This high sensitivity, compact size, and online detection scheme give the magnetic-vector sensor significant potential for applications involving the detection of weak magnetic-field vectors.

## Figures and Tables

**Table 1:** Comparisons between the smartphone-based and the traditional platforms.

SPR Scheme	Simulation/Experiment	Vector	Sensitivity	Reference
Prism	Simulation	No	0.061°/Oe	[28]
Side-hole fiber	Simulation	No	1.063 nm/Oe	[29]
D-shaped PCF	Simulation	No	0.087 nm/Oe	[30]
Metal-dielectric-Metal	Simulation	No	0.027 nm/Oe	[42]
Tapered fiber	Experiment	No	~1.0 nm/Oe	[31]
No-core fiber	Experiment	No	0.303 nm/Oe	[32]
Tilted FBG	Experiment	Yes	0.18 nm/Oe 2 nm/°	[21]
Side-polished MMF	Experiment	Yes	0.599 nm/Oe −5.63 nm/°	[33]
Side-polished FMF	Experiment	Yes	0.692 nm/Oe −11.917 nm/°	This work
